# Repeated Artemisinin-Based Combination Therapies in a Malaria Hyperendemic Area of Mali: Efficacy, Safety, and Public Health Impact

**DOI:** 10.4269/ajtmh.2012.11-0649

**Published:** 2012-07-01

**Authors:** Issaka Sagara, Bakary Fofana, Jean Gaudart, Bakary Sidibe, Amadou Togo, Sekou Toure, Kassim Sanogo, Demba Dembele, Alassane Dicko, Roch Giorgi, Ogobara K. Doumbo, Abdoulaye A. Djimde

**Affiliations:** Malaria Research and Training Center, Department of Epidemiology of Parasitic Diseases, Faculty of Medicine, Pharmacy and Odonto-Stomatology, University of Bamako, Bamako, Mali; Aix-Marseille University, Marseille, France

## Abstract

Artemisinin-based combination therapies (ACTs) are the first-line treatment of uncomplicated malaria. The public health benefit and safety of repeated administration of a given ACT are poorly studied. We conducted a randomized trial comparing artemether-lumefantrine, artesunate plus amodiaquine (AS+AQ) and artesunate plus sulfadoxine-pyrimethamine (AS+SP) in patients 6 months of age and older with uncomplicated malaria in Mali from July 2005 to July 2007. The patient received the same initial treatment of each subsequent uncomplicated malaria episode except for treatment failures where quinine was used. Overall, 780 patients were included. Patients in the AS+AQ and AS+SP arms had significantly less risk of having malaria episodes; risk ratio (RR) = 0.84 (*P* = 0.002) and RR = 0.80 (*P* = 0.001), respectively. The treatment efficacy was similar and above 95% in all arms. Although all drugs were highly efficacious and well tolerated, AS+AQ and AS+SP were associated with less episodes of malaria.

## Introduction

According to the World health Organization (WHO) 2010 report, in the 106 malaria-endemic countries, 225 million cases of malaria led to an estimate of 781,000 deaths in 2009.^1^ Once a person develops malaria, the only means of reducing suffering and preventing death is by diagnosing and treating the disease.^2^ To counter the threat of resistance of *Plasmodium falciparum* to monotherapies, and to improve treatment outcome, WHO recommends that artemisinin-based combination therapies (ACTs) be used for the treatment of uncomplicated *P. falciparum* malaria.^2^

The choice of the ACTs in sub-Saharan African countries was mostly based on the efficacy and safety data of a single malaria episode treatment that does not take into account the long-term impact on malaria incidence and safety, which may have a given ACT when used over and over to treat consecutive malaria episodes.

In addition, as the first generation ACTs are being rolled out, studies show that they may rapidly increase the prevalence of molecular markers associated with resistance to the partner drugs, i.e., lumefantrine, amodiaquine, or sulfadoxine-pyrimethamine.^3^–^7^

Although data are available for the use of ACTs during single-episode treatment,^8^–^12^ there are limited data on safety and public health benefits in terms of overall incidence of clinical episodes during the use of the same ACT during repeated malaria episodes treatment.^13^–^17^

To address these gaps this study aimed to evaluate the public health impact in terms of incidence of clinical episodes and the efficacy and safety of artemether-lumefantrine (AL), artesunate plus amodiaquine (AS+AQ), and artesunate plus sulfadoxine-pyrimethamine (AS+SP) when used repeatedly for consecutive clinical malaria episodes during as long as 24 months.

## Materials And Methods

### Study site.

The study was conducted from July 2005 to July 2007 in Bougoula-Hameau, a peri-urban village of ∼5,000 inhabitants located in the South of Mali. *Plasmodium falciparum* is hyperendemic with seasonal peaks that occur during the rainy season (June–November).

### Study design and procedures.

This was an open-label, randomized clinical trial comparing three different ACTs: AL, AS+AQ, and AS+SP.

When malaria was suspected finger-prick blood was taken for thick blood films. After ensuring that inclusion criteria are met, a written informed consent was obtained. Patients were included if they were at least 6 months of age, weighed ≥ 5 kg, resided in the study village, were able to receive oral treatment, had an axillary temperature ≥ 37.5°C, and had *Plasmodium* sp. infection with a parasite density between 2,000 and 200,000 asexual forms per microliter of blood for the first episode. The first subject was enrolled in July 2005 and the enrollment completed with the last subject enrolled 30 October 2006, although the last malaria episode follow-up ended in July 2007. Subjects with the following characteristics were not included in the study: the presence of severe or complicated malaria^18^; a severe concomitant pathology or one that needed a medical follow-up incompatible with the study, an allergy to one of the drugs involved in this study, and a pregnancy.

Eligible subjects were randomized to receive either AL or AS+AQ or AS+SP, and followed up for 28 days after the treatment initiation with one of the three ACTs, according to the 2003 WHO^19^ treatment assessment guideline. Block-randomization was used to allocate the patients to the three treatment arms. Treatment assignments were through sequentially numbered opaque envelops that concealed the actual treatment to which the patient was randomized.

Once subjects were assigned to a given group, in the event of subsequent uncomplicated malaria episodes defined as the presence of malaria signs or symptoms plus *Plasmodium* sp. infection (a positive blood smear with parasite density < 200,000 parasites/μL), they were re-treated with that same treatment regimen (except in case of parasite recurrence cases occurring within each 28-day treatment follow-up period where quinine was used). Patients were closely followed both clinically and biologically to record any adverse event. Patients with > 200,000 parasites/μL or who had treatment failure during the 28-day follow-up period or who developed severe malaria were treated with quinine and/or hospitalized if necessary. The study team was present 24 hours a day, 7 days a week at the study site during the study period.

Enrolled patients were asked to return for clinical and/or biological follow-up on Days 1, 2, 3, 7, 14, 21, 28, or on days of recurrent illness. Patients or guardians were asked about any medicine consumption outside the research facility since the last clinic visit. At each visit, a physical examination, including axillary temperature, was performed and blood was taken for thick smears. Patients were asked to return to the clinic any time if they became ill.

Thick blood films and blood onto filter paper samples were made on Days 0, 2, 3, 7, 14, 21, 28, and on any days of recurrent illness. Parasitemia were measured by counting the number of asexual parasites against 300 leukocytes on the thick blood film, and then reported to a microliter of blood based on a count of 7,500 leukocytes/μL.^20^ If gametocytes were seen, a gametocyte count was performed against 1,000 leukocytes. Although this was an open-label study, all malaria slides were read in a blinded fashion by the technicians, because the parasitological result was the key element in the determination of the primary endpoint.

Blood blotted onto filter-paper samples were obtained for molecular analyses of drug resistance markers and to distinguish recrudescent from re-infections.

Hematology (hemogramme) and biochemistry (creatinemia and alanine aminotransferase) analysis were performed on Day 0, 7, and also on any other day if deemed clinically necessary to assess the biological toxicity of the study drugs. Normal values were based on laboratory parameters obtained from Malian population (our unpublished data). The values outside the normal ranges were considered abnormal and graded.

MSP1, MSP2, and CA1 polymorphisms were analyzed using standard methods with polymerase chain reaction (PCR) to discriminate true recrudescent parasites from new infections.^7^

Severe malaria cases and parasite recurrence cases occurring within each 28-day treatment follow-up period were treated with quinine (8 bmg/kg three times daily for 7 days) and re-enrolled (after complete remission) for the subsequent uncomplicated malaria (presence of malaria signs or symptoms plus *Plasmodium* sp. infection, which is defined as a positive blood smear with parasite density < 200, 000 parasites/μL) with the same study treatment arm. Quinine was chosen because it was the second-line drug for malaria treatment.

The efficacy data analysis was based on the WHO 2003, 28-day treatment efficacy assessment guideline^19^; the efficacy was determined in each subject after an uncomplicated malaria episode where the subject received the same ACT as during the initial randomization study arm. Treatment outcomes were classified as early treatment failure (ETF), late clinical failure (LCF), late parasitological failure (LPF), and adequate clinical and parasitological response (ACPR).

All study medicines were administrated orally by designated study personnel at the health center. The AS+AQ was Arsucam coblister (AS 50 mg/AQ 153 mg; Sanofi-Aventis, Paris, France), given once daily for 3 days to the following scheme: for patients < 10 kg, 1/2 tablet AS + 1/2 tablet AQ; 10–20 kg, 1 tablet AS+ 1 tablet AQ; 21–40 kg, 2 tablets AS+ 2 tablets AQ; > 40 kg, 4 tablets AS+ 4 tablets AQ.

The AS+SP was Arsumax (AS 50 mg; Sanofi-Aventis, Paris, France) + sulfadoxine–pyrimethamine (S _ 500 mg/P _ 25 mg, Fansidar Roche, Burlington, NC), given once daily for 3 days to the following scheme: for patients ≤ 10 kg, 1/2 tablet AS+ 1/2 tablet SP; 11–20 kg, 1 tablet AS+ 1 tablet SP; 21–40 kg, 2 tablets AS+ 2 tablets SP; > 40 kg, 4 tablets AS+ 3 tablets SP. The SP is given only the first day, whereas AS is given over 3 days.

The AL was Coartem (Novartis Pharma AG, Basel, Switzerland), given twice daily for 3 days to the following scheme: for patients < 15 kg, 1 tablet; 15–24 kg, 2 tablets; 25–34 kg, 3 tablets; ≥ 35 kg, 4 tablets.

### Ethics.

The study protocol was reviewed and approved by the Ethical Committee of the Faculty of Medicine, Pharmacy and Dentistry, University of Bamako, Mali. Community permission was obtained before the start of the study as described by Diallo and others.^21^ Individual, written, informed consent was obtained from each patient (if adult) or parent or guardian of each child before enrolment.

### Sample size.

Calculation of sample size was based on the assumptions that patients experienced an average of two clinical episodes per person per year if treated by AL and living in the study areas and that treatment with AS+AQ or AS+SP would reduce this value by 35%, which is 1.3 clinical episodes per person per year. With an error risk alpha set at 0.05, a power of 90%, 174 subjects were needed in each arm. If one takes into account loss of follow-up and data attrition, 260 subjects were needed in each arm (total sample of 780).

### Data management and statistical procedures.

Data were double entered and validated using Microsoft Access (Microsoft, Redmond, WA) and then analyzed using Stata software version 10.0 (StataCorp., College Station, TX).

For univariate analysis, χ^2^ or Fisher's test if appropriate was performed to compare categorical variables. Normally distributed continuous variables were compared with analysis of variance. Nonparametric tests were used when appropriate.

Parasite recurrence occurring within each 28-day treatment follow-up was counted as a new malaria episode because it was associated with full malaria rescue treatment regimen. However, following 14 days after each malaria rescue treatment with quinine, the subject was not considered at risk of malaria, therefore that time was deducted in the subject's total length of time. Negative binomial regression analysis adjusted for repeated events for the same subject was used to estimate relative risk between study drugs.

The incidence rate is defined as number of malaria episodes in each study arm divided by the time length in days of each participant (person-time) and then expressed in a year (by dividing by 365.25 days).

### Efficacy analysis concerned all combined malaria episodes from each participant.

An intention-to-treat (ITT) analysis for drug efficacy was based on all subjects randomized to their respective treatment arm.

A per-protocol (PP) analysis for drug efficacy was also performed and excluded missing (lost to follow-up, withdrawal) data and major protocol violation cases.

For safety data, all subjects who received at least one dose starting from their first episode of malaria were included into the safety analysis.

## Results

### Study trial profile and baseline characteristics.

**Figure 1.**
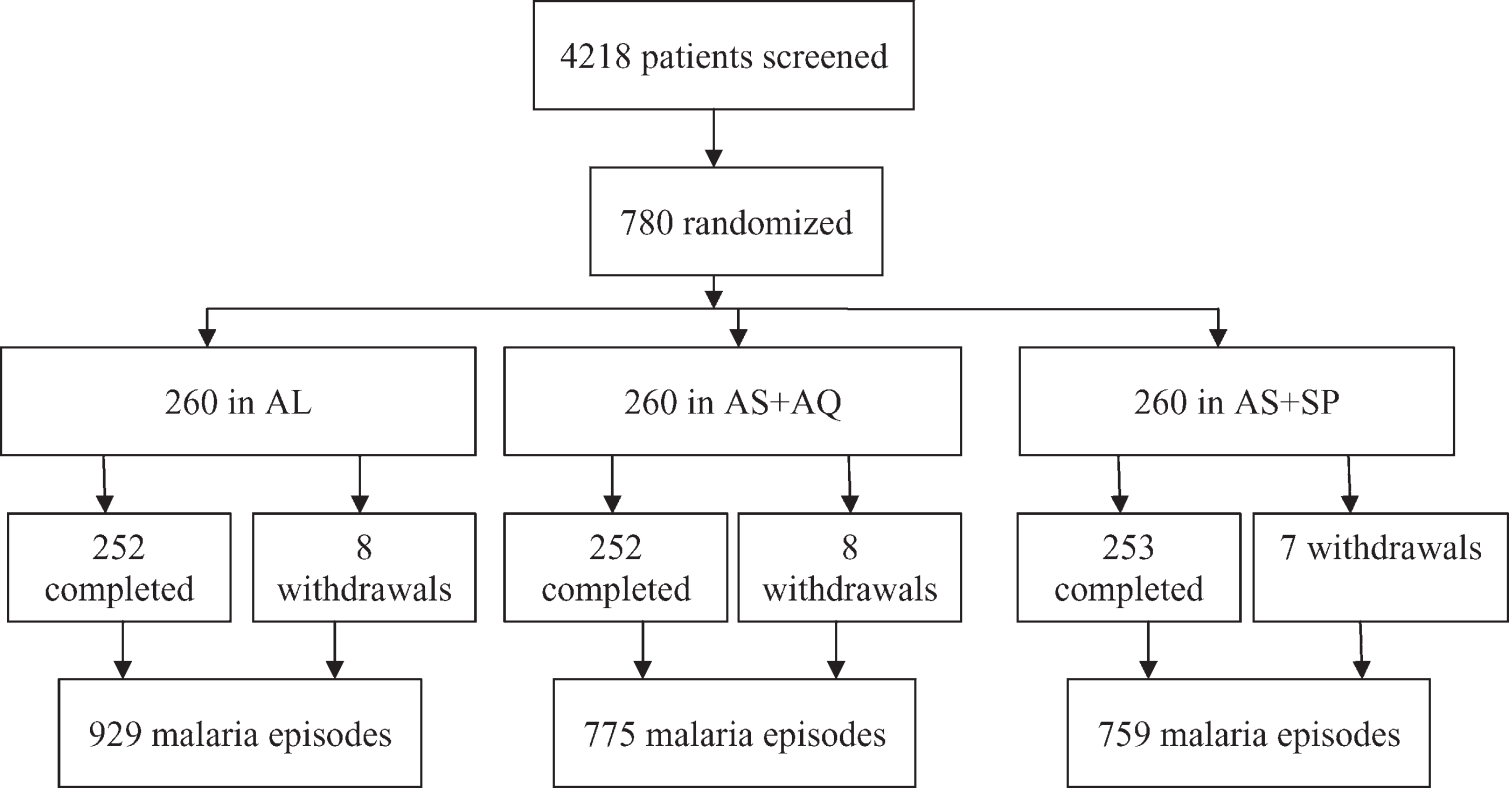
Study trial profile.

The trial profile is summarized in Figure 1. A total of 4,218 patients attended the clinic with malaria symptoms, 780 subjects who met the enrollment criteria were included in the study; 260 patients per treatment arm. A total of 2,463 malaria episodes were documented during the study, although 23 participants did not complete the study (Figure 1). The reasons for not completing the study were: travel (2 in the AL arm, 4 in the AS+AQ arm, and 3 in the AS+SP arm); consent withdrawal (4 in the AL arm, 1 in the AS+AQ arm, and 1in the AS+SP arm); treatment allocation errors that were not excluded from the study but adjusted into the analysis (1 subject received AS+AQ instead of AS+SP the original allocated treatment arm, 1 subject received AS+SP instead of AS+AQ the originally allocated treatment arm, 1 subject received AS+SP for the second episode instead of AL the originally allocated treatment arm); 2 cases of death in AS+SP arm;1 case in the AL arm received chloroquine treatment at home during the first malaria episode treatment; 1 case in the AS+AQ arm received quinine treatment at home during the second malaria episode treatment; and 1 case in the AS+AQ arm developed severe anemia during its first malaria episode treatment that required blood transfusion at the hospital.

At enrollment (Table 1), the mean age, sex ratio, the median parasitemia of *P. falciparum*, and the prevalence of gametocyte carriage were all similar in all three treatment arms. Only two cases of *Plasmodium ovale* and no case of *Plasmodium malariae* monospecific infection was detected, whereas 8% (*N* = 780) of *P. falciparum* + *P. malariae* and 0.8% (*N* = 780) of *P. falciparum* + *P. ovale* mixed infection were found (data not shown).

### ACT and malaria incidence rate.

Table 2 shows that the malaria incidence rate per person and per year was 2.70 (95% confidence interval [CI]: 2.53–2.88), 2.25 (95% CI: 2.12–2.44), and 2.18 (95% CI: 2.06–2.38) respectively in AL, AS+AQ, and AS+SP arms.

In multivariate modeling (Table 2), patients in the AS+AQ and AS+SP arms had significantly less risk of having malaria episodes than patients in the AL arm; risk ratio [RR] = 0.84 (*P* = 0.002) and RR = 0.80 (*P* = 0.001), respectively. Therefore, compared with AL the risk of malaria incidence reduction was 16% and 20% for AS+AQ and AS+SP, respectively. Using the age group ≥ 15 years of age as reference (Table 2), patients in the age group 10–14 years, the age group 5–9 years, and the age group < 5 years had significantly more risk of having malaria episodes; RR = 1.60 (*P* = 0.002), RR = 2.44 (*P* = 0.001), and RR = 2.76 (*P* = 0.001), respectively.

### ACT efficacy on uncomplicated malaria.

Non-PCR-corrected ACPR was significantly different between the study arms (62.0% (*N* = 665), 78.5% (*N* = 619), and 89.1% (*N* = 678) in AL, AS+AQ, and AS+SP arms, respectively, *P* < 0.001).

**Figure 2.**
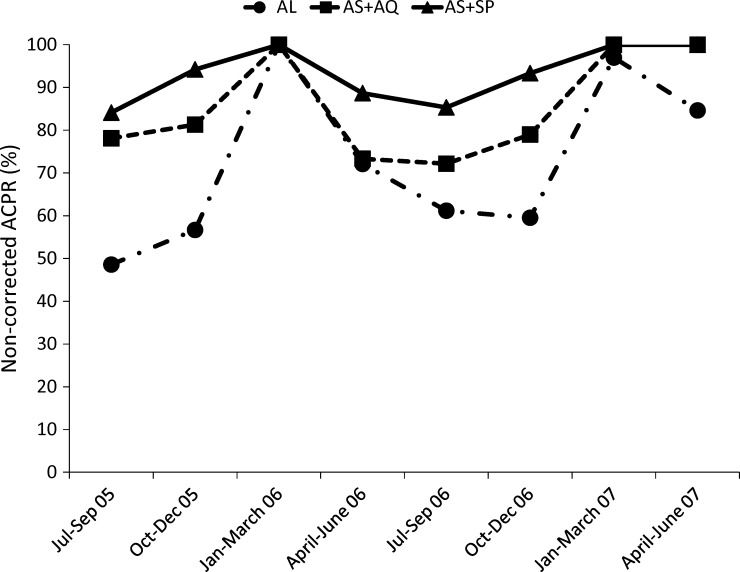
Per protocol non-polymerase chain reaction (PCR) corrected adequate clinical and parasitological responses (ACPRs) per study arm, per season, and per year. Non-PCR corrected ACPRs per study arm, per season, and per year. The numbers of patients (n/*N*) with non-corrected ACPRs were 51/105, 82/105, and 90/107, respectively, for AL, AS+AQ, and AS+SP in July–September 2005, 68/120, 87/107, and 114/121 in October–December 2005; 29/29, 25/25, and 22/22 in January–March 2006; 31/43, 33/45, and 47/53 in April–June 2006; 115/188, 127/176, and 163/191 in July–September 2006; 75/126, 94/119, and 140/150 in October–December 2006; 32/33, 24/24, and 16/16 in January–March 2007; 11/13, 13/13, and 12/12 in April–June 2007.

Figure 2 shows that there was a significant difference in the non-PCR-corrected ACPRs between the treatment arms according to the season. The lowest ACPRs were documented during rainy seasons when malaria transmission is highest (July–September followed by October–December). This seasonal variation in the efficacy of ACTs was documented for two consecutive years.

Using the ITT analysis (Table 3), the PCR-corrected ACPR were high and similar for all study arms 94.4%, 95.8%, and 97.9% in the AL, AS+AQ, and AS+SP arm, respectively. The difference of efficacy between any two arms treatment is < 5%.

Using the PP analysis (Table 3), the PCR-corrected ACPR were also high for all study arms; 99.1%, 98.7%, and 99.6% respectively in the AL, AS+AQ, and AS+SP arm.

### Safety and tolerability.

The adverse events with the highest incidence was mild vomiting, which was higher in AS+AQ and AS+SP than in the AL arm (*P* < 0.001), followed by abdominal pain and headaches (Table 4), which was similar in the three arms. One subject who had vomiting in the AS+AQ arm discontinued the oral treatment and parenteral quinine was provided because of the repeated drug dose vomiting. The frequency of the other adverse events was similar in the three arms. The incidence of laboratory adverse events was similar in the three treatments arms except for the creatinemia which significantly different; 5.09%, 6.33% and 15.19% in the AL, AS+AQ, and AS+SP arm respectively, *P* < 0.001.

Serious adverse events (SAEs) were documented and included 2 severe cases of anemia (1 in AL arm, 1 in AS+AQ arm); 2 cases of seizure (1 in AL arm, 1 in AS+AQ arm); 1 case of severe malaria (in AL arm); 1 case of coma caused by hypoglycemia (in AS+SP); 1 case of presumed pneumopathy (in AS+SP arm); 2 cases of death in AS+SP arm (one caused by pneumonia and the other to hypoglycemia). The occurrence of SAE was comparable in the three arms. All of the above non-fatal SAE resolved without sequelae. The two cases of death happened in the AS+SP arm, although a causal relationship with the study product was not established. All SAEs were assessed as not related to the study drugs. There was no significant laboratory abnormalities (hemogramme, creatinemia, and alanine aminotransferase parameters) related to any of the study treatment drugs (Table 4).

## Discussion

Over 24 months of follow-up of this study show that the incidence of uncomplicated malaria was significantly lower in the AS+SP and AS+AQ arms than in the AL arm. This could be explained by the lowest re-infection rate seen in the AS+SP and AS+AQ arms and maybe because of the longer half life of the partner drugs^7^ (AQ or SP) or else to increasing resistance to lumefantrine. However, *in vitro* studies conducted during the same period in Mali did not show any lumefantrine resistance.^22^

Our results are in contrast with the result of a study conducted in Ghanaian children^15^ with repeated treatment of AS+AQ or AL where a similar incidence rate between the two study arms was reported. This may be because in our study, we counted late parasitological failures caused by re-infections (as determined by PCR) as new malaria episodes. As in our study, the author reported good safety profile and high 28-day follow-up treatment efficacy in both arms (AS+AQ and AL). We found that malaria incidence was high in children < 5 years of age but also in children > 5 years of age compared with adults.

The high PCR-corrected 28-day follow-up treatment efficacy results are consistent with reports from the same area in
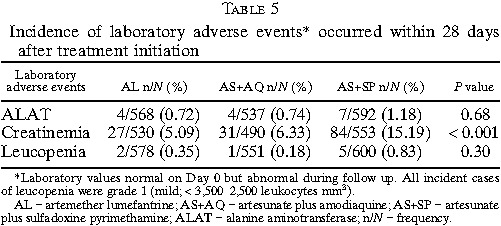
2004 where ACPRs rates of 100% and 99.1% were found with AS+SP and AS+AQ, respectively.^7^ Our data are similar to those of Senegal^17^ and Indonesia with AS+SP^23^ and a multicenter study in Africa with AL and AS+AQ.^24^

The re-infection rate was higher in the AL arm as compared with AS+AQ and AS+SP arms (*P* < 0.001). This high reinfection rate with AL is in contrast with studies in Uganda^25^ and Tanzania^13^ where lower reinfection rates were reported in AL compared with AS+AQ. The authors thought that the higher reinfection rates in AS+AQ could be explained by the relatively high amodiaquine resistance level in the study area. This study found that the risk of recurrent parasitemia (uncorrected PCR 28-day follow-up ACPR) malaria had a significant seasonal variation. We found significant recurrent parasitemia during the rainy season compared with the dry season. This study finding is consistent with a Ugandan study,^25^ which also found significant seasonal variation in recurrent parasitemia despite the perennial malaria transmission pattern in that study setting. The significant recurrent parasitemia during the rainy season compared with the dry season could be explained by the high intensity of the malaria transmission during the rainy season compared with the dry season.

Our participants received quinine as a second-line treatment. However, it is unclear whether quinine, the same ACT, or a different ACT would be the optimal treatment. Given that nearly all recurrent parasitemia were caused by new infections, it might have been reasonable to retreat with the same ACT regimen, rather than with quinine.

The repeated treatment using the same ACT in this study showed a good safety profile. This finding is consistent with other studies that also reported a good safety profile of repeated treatment of ACT,^13^–^15^,^17^ even in chronically malnourished children with or without HIV infection.^14^ Although AL was associated with a higher reinfection rate and hence higher malaria incidence rate, it did produce fewer side effects such as vomiting.

Limitations of this study include the consideration of asymptomatic parasite carriers from Day 7 during each 28-day treatment follow-up as treatment failures (as in the WHO treatment assessment guidelines^19^), which were treated with quinine and counted as malaria episodes for new infections as determined by PCR. The overall malaria episodes may be similar if one considered only symptomatic parasite recurrence during each 28-day treatment follow-up and the clinical episodes occurring afterward. Although our sample size calculation assumptions expected differences equal or greater than 35%, we observed statistically significant differences in malaria incidence reduction of 16% and 20% with AS+AQ and AS+SP, respectively, compared with AL. This is probably because our original calculation was conservative and yielded a rather large sample size, resulting in smaller differences being statistically significant. In addition, the mean malaria incidence was higher than expected (two episodes expected but almost three episodes observed)

We show that ACTs that had very high and similar efficacy and safety by the standard 28-day drug efficacy test could have a different impact on malaria incidence over a longer period. In this era of high efficacy of ACTs, the choice of a first-line treatment policy should go beyond short-term efficacy and safety to include longer follow-ups and repetitive treatments with the same regimen. Additional studies will be required to assess the overall impact of the large-scale deployment of ACTs on malaria in the field.

## Figures and Tables

**Table 1:** Baseline characteristics per treatment group and overall at enrollment*

Characteristics	Regimen (AS+AQ)(*N* = 260)	Regimen (AS+SP)(*N* = 260)	Regimen (AL)(*N* = 260)	*P* value
Age (years)				
Mean ± SD	5.37 ± 6.19	5.23 ± 4.1	5.94 ± 6.8	0.51
Median (min, max)	4.1 (0.5,68.6)	4.3(0.5,30.5)	4.3(0.5,46.6)	
Age category n (%)				0.72
< 5	155 (59.6)	147 (56.5)	146 (56.2)	
5–9	78 (30.0)	85 (32.69)	88 (33.9)	
10–14	18 (6.9)	21 (8.1)	14 (5.4)	
≥ 15	9 (3.5)	7 (2.7)	12 (4.6)	
Gender – n (%)				
Female	142 (54.6)	129 (49.6)	130 (50.0)	0.45
*P. falciparum* (/μL)				
Median (Q1,Q3)	21,550 (10,375, 45,150)	23,225 (9,990, 44,190)	21,925 (10,440, 42,050)	0.99
Gametocyte– n (%)	16 (6.2)	18 (6.9) 28 (10.8)	0.11	

* n = frequency; Q1 = 25th percentile; Q3 = 75th percentile; Min = minimum; Max = maximum; AL = artemether-lumefantrine; AS+AQ = artesunate plus amodiaquine; AS+SP = artesunate plus sulfadoxine-pyrimethamine.

**Table 2:** Incidence rates and RR per treatment arm and per age (in year) category*

Factors	Numbers of episodes (years at risk)	Incidence rate (95% CI)	Adjusted* RR (95% CI)	*P* value
AL	923 (341.42)	2.70 (2.53–2.88)	1	
AS+AQ	768 (340.59)	2.25 (2.12–2.44)	0.84 (0. 75–0.93)	0.001
AS+SP	756 (346.99)	2.18 (2.06–2.38)	0.80 (0.72–0.89)	< 0.001
Age ≥ 15	40 (41.07)	0.97 (0.70–1.33)	1	
Age 10–14	108 (71.44)	1.51 (1.24–1.83)	1.59 (1.07–2.34)	0.020
Age 5–9	779 (337.49)	2.31 (2.15–2.48)	2.41 (1.72–3.38)	< 0.001
Age < 5	1520 (579.0)	2.63 (2.49–2.76)	2.74 (1.96–3.82)	< 0.001

* Adjusted for age.

RR = risk ratio; AL = artemether-lumefantrine; AS+AQ = artesunate plus amodiaquine; S+SP = artesunate plus sulfadoxine-pyrimethamine; CI = confidence interval.

**Table 3:** Efficacy evaluation on Day 28 after PCR correction* ITT and PP analysis

	AL (*N* = 665)	AS+AQ (*N* = 619)	AS+SP (*N* = 678)	*P* value
Possible failure* – n (%)	31 (4.7)	18 (2.9)	11 (1.6)	0.005
ETF† – n (%)	0 (0)	1 (0.2)	0 (0)	0.32
LCF† – n (%)	2 (0.3)	1 (0.2)	0 (0)	0.43
LPF† – n (%)	4 (0.6)	6 (1.0)	3 (0.4)	0.5
ITT–ACPR – n (%)	628 (94.4)	593 (95.8)	664 (97.9)	0.004
PP–ACPR – n/*N* (%)	628/634 (99.1)	593/601 (98.7)	664/667 (99.6)	0.25
Reinfection rate – n/*N* (%)	231/634 (36.4)	117/601 (19.5)	62/667 (9.3)	< 0.001

* Possible failure: lost to follow-up or withdrawal or subjects for which filters were missing or undetermined PCR.

† These are computed according to ITT analysis.

PCR = polymerase chain reaction; ITT = intention-to-treat; PP = per protocol; AL = artemether-lumefantrine; AS+AQ = artesunate plus amodiaquine; AS+SP = artesunate plus sulfadoxine-pyrimethamine; n/*N* = frequency; ETF = early treatment failure; LCF = late clinical failure; LPF = late parasitological failure; ACPR = adequate clinical and parasitological failure.

**Table 4:** Incidence of adverse events* occurred within 3 days after treatment initiation

Symptoms/signs	AL n/*N* (%)	AS+AQ n/*N* (%)	AS+SP n/*N* (%)	*P* value
Vomiting	25/281 (8.9)	58/269 (21.6)	67/289 (23.2)	< 0.001
Headaches	2/30 (6.7)	7/39 (18.0)	5/49 (10.2)	0.36
Asthenia	0/113	1†/123 (0.81)	0/135	0.64
Dizziness	0/129	2/132 (1.5)	1/146 (0.7)	0.53
Anorexia	0/109	3/123 (2.4)	2/140 (1.4)	0.33
Abdominal pain	8/100 (8.0)	8/85 (9.4)	9/104 (8.7)	0.94
Rash	1/654 (0.15)	0/612	0/666	0.65

* These adverse events are considered to be related to the study drugs because of the temporal relationship with the study drug although the possibility that these symptoms can also be caused by malaria.

† The subject was having difficulty walking during the asthenia episode.

AL = artemether-lumefantrine; AS+AQ = artesunate plus amodiaquine; AS+SP = artesunate plus sulfadoxine-pyrimethamine; n/N = frequency.
